# An evaluation of rational prescribing in hospital outpatient practice in Sierra Leone and assessment of affordability of a prescription as an outcome

**DOI:** 10.11604/pamj.2018.31.174.16729

**Published:** 2018-11-13

**Authors:** Christine Princess Cole, Philip Routledge

**Affiliations:** 1 Department of Clinical Pharmacy and Therapeutics, Faculty of Pharmaceutical Sciences, College of Medicine and Allied Health Sciences, University of Sierra Leone, Sierra Leone; 2 Pharmacology, Therapeutics & Toxicology, School of Medicine, Cardiff University, Wales, United Kingdom; 3 All Wales Therapeutics and Toxicology Centre, University Hospital Llandough, Penarth, Wales, United Kingdom

**Keywords:** Rational prescribing, cost, affordability

## Abstract

**Introduction:**

Medicines are the most frequently used intervention in healthcare. Rational and cost-effective prescribing is especially important in countries where access to effective medicines may be challenged by affordability issues. This study describes the prescribing patterns of doctors in government hospitals in Freetown, Sierra Leone, considering the scope for rationalising prescribing and reducing cost to the patient.

**Methods:**

A descriptive, retrospective, cross-sectional study was conducted at four hospitals, using selected World Health Organisation (WHO) indicators applied to 600 prescriptions, after systematic random sampling. The data was analysed using SPSS.16 and the Index of Rational Drug Prescrib-ing (IRDP) calculated. The Spearman's rank coefficient was used to examine possible associations between the number of medicines prescribed as generics and from the National Essential Medicines List (NEML) and cost of the prescription respectively. Affordability was determined from the average number of days of work required to purchase a prescription, based on the minimum wage of the lowest paid government worker in Sierra Leone.

**Results:**

The mean number of medicines per prescription from the four hospitals was 4.37(range 4.18-4.56) with 57% prescribed generically and 64% from the NEML. An antibiotic and injection were found on 72% and 26% of prescriptions respectively. The overall IRDP was 2.65/5. The aver-age cost per prescription was Le. 29,376.30 ($6.78), equivalent to 43 days of work of the lowest paid government worker.

**Conclusion:**

In this study, opportunities were identified for significant rationalisation and improvement in cost-effective prescribing.

## Introduction

Medicines are the most frequently used intervention in healthcare [1] with 74% of hospital outpa-tient visits involving their administration [2]. Like immunization for common childhood diseases, the appropriate use of medicines is one of the most cost-effective components of health care [3]. However, opportunities are sometimes missed, with over half of them sometimes being prescribed, dispensed or sold inappropriately [4]. Rational prescribing involves maximising clinical and cost-effectiveness, minimising harm, while respecting patient choice [5-7]. Its importance cannot be overemphasised in developing countries like Sierra Leone with only 0.2 physicians per 10,000 population [8] and non-comprehensive health insurance systems. The World Health Organisation (WHO) drug use indicators are used to identify and describe problems with medicines use in health facilities [9]. Specifically, the WHO prescribing indicators are the average number of medicines prescribed per patient encounter, percentage of medicines prescribed by generic name, percentage of encounters with an antibiotic and injection prescribed respectively and the percentage of medicines prescribed from the National essential medicines list (NEML) [9]. Polypharmacy is the simultaneous use of several medicines by a patient, or prescribing too many inappropriate drugs that have no legitimate indication [10, 11]. Prescription of generic medicines is being promoted because they are less expensive than branded drugs, providing considerable cost savings and the risk of error when dispensing and administering them to patients is reduced, as each medicine has only one generic name [12]; the proportion of generic prescribing can be used as an indicator of efficient prescribing practice [12]. Inappropriate use of antibiotics accelerates the emer-gence and spread of antimicrobial resistance (AMR) and the more frequently, and the greater the used, the faster the microorganisms they target can evolve into resistant strains [13]. Unnecessary use of injections increases treatment costs and can potentially cause infection with blood borne pathogens if needle stick injuries arise [14]. They have been noted to be associated with rational prescribing and inevitably, prescribing costs [14, 15].

An initial obstacle to the use of the WHO indicators was the lack of reference standard values which would serve as yardsticks for comparison when assessing prescribing practices and evaluating interventional and supervisory efforts. The absence of such reference values for these indices made arguments for rational use of drugs abstract and difficult to appreciate [16]. Based on these indicators, the Index of Rational Drug Prescribing (IRDP) provides a comparative standard to as-sess rational prescribing performance [16]. An integral component of rational medicines use is minimising cost to patients [17]. Medical insurance schemes cover less than 8% of the population in Africa, making out-of-pocket payments the main source of health care financing [3]. Thus, affordability of medicines strongly influences access to treatment [3]. In Sierra Leone, the national free health care insurance scheme only covers children aged less than 5 years, pregnant women and lactating mothers [18]. With about one-third of the population living in the capital city Freetown and its environs, and over three-quarters of them living below the nationally defined poverty line [8], many patients seek care at government/public hospitals in Freetown at their own expense. Thus rational prescribing at these hospitals is vitally important. In 1997, Lisk and Palmer [19], assessed the prescribing patterns of doctors and dispensers in the Western Area (Freetown and its environs) of Sierra Leone using the WHO indicators and concluded that it was mostly irrational. They however did not use a standardised tool to measure rational pre-scribing and did not determine the impact on affordability. This study therefore aims not only to describe the current prescribing practices of doctors in government hospitals in Freetown, Sierra Leone, using the WHO drug indicators, but to also assess the degree of rational prescribing and de-termine the relationship of such prescribing on patients' ability to afford prescribed medicines.

## Methods

**Study setting:** Sierra Leone is administratively divided into the Western Area (including Freetown, the capital city which is densely populated) and the Northern, Southern and Eastern provinces. The four hospitals selected were Connaught Hospital (the tertiary referral hospital for general medical and surgical cases in Freetown) and three secondary hospitals (Rokupr Government Hospital in the eastern part of Freetown, Macauley Street Government Hospital in the central part of Freetown and Lumley Government Hospital in the western part of Freetown). The individual hospitals are anonymised and their data randomised in order in the tables.

**Ethical approval:** Ethical clearance was obtained from the Sierra Leone Ethics and Scientific Review Committee and informed consent was obtained from each hospital administration.

**Study design:** A descriptive, retrospective, longitudinal study was conducted. Using the Leslie Kish formula for sample size calculation (using 50% proportion and 4% degree of precision), a total of 600 prescriptions were required to be sampled. This number was equally divided between the four health facili-ties, resulting in 150 prescriptions being sampled at each facility. The sampling frame included all patients who presented at the four study sites during the study period. At the selected hospitals, all outpatient treatment charts or prescription records within the study period were arranged by dates (August 2012 to July 2013) and those used for the study selected by systematic random sampling. This ensured that bias due to possible seasonal variations affecting disease patterns and therefore prescribing was minimised. A structured data collection tool was used to collect data on patient demographics (age and sex) and five selected prescribing indicator values from prescriptions. The cost per prescription was al-ready stated on some prescriptions or calculated from the price list of medicines obtained from the hospital pharmacist. Patients aged less than 5 years old and pregnant women were excluded from the study as they benefit from a free health care insurance scheme.

**Data analysis:** The data for each WHO prescribing indicator was calculated and analysed using the Statistical Pack-age for Social Sciences (SPSS) Version 16. From these results obtained, the IRDP, using the meth-od developed by Dong and colleagues [16] was calculated. For the purposes of this study, polypharmacy was defined as a prescription with five or more medicines and the index of polypharmacy was measured by the percentage of non-polypharmacy prescriptions. The index of generic prescribing was measured by the percentage of medicines prescribed by generic name. The index of rational antibiotic prescribing was calculated by dividing the optimal level (30%) by the percentage of prescriptions with an antibiotic. The index of safe injection prescribing was calculated by dividing the optimal level (10%) by the percentage of prescriptions which had an injection. The essential medicines prescribing index (EMPI) was also measured by the percentage of drugs prescribed that were on the National Essential Medicines List. Each indicator has an optimal index of 1 and the closer to 1 the calculated value is, the more ration-al, the prescribing van be considered (Table 1). The IRDP is then calculated by adding together the values of the five indices [16] (the maximum value is thus 5). The IRDP for each hospital and overall IRDP were then calculated. To determine if there was any association between cost per prescription (as the dependent variable) and the percentage of medicines prescribed by generic name and prescribed from the national essential medicines list (as independent variables), the Spearman's rank coefficient was calculated by correlating each variable and cost. One reasonable method of expressing medicine affordability is in terms of the days' wages that a country's Lowest Paid unskilled Government Worker would require to spend on a standard course of treatment [20, 21]. To assess the extent of affordability of a prescription, the average cost of the medicines prescribed was calculated and the number of working days required to purchase this was determined based on the daily wage earned by the lowest paid unskilled government worker.

**Table 1:** Optimal levels of drug prescribing indicators

Prescribing indicators	Optimal level (%)	Optimal index
Percentage of nonpolypharmacy prescriptions	≤3	1
Percentage of drugs prescribed by generics	100	1
Percentage of prescriptions including antibiotics	≤30	1
Percentage of prescriptions including injections	≤10	1
Percentage of drugs prescribed from EML or formulary	100	1

EML: Essential medicines list

## Results

Over half of the patients (63%) were aged between 5 and 40 years and more female (53%) than male (47%) patients were seen at the four hospitals overall (Table 2). There were on average 4 medicines (range 3-7) per prescription, with 57% of them prescribed as generics and over 60% from the NEML (Table 3). Antibiotic prescribing for outpatients at gov-ernment hospitals in Freetown is very high, with 76% of prescriptions involving an antibiotic (Table 3). Injections were also prescribed for 26 % of outpatients (Table 3). The overall mean IRDP was 2.65. The highest indices were those for polypharmacy (0.64) and essential medicines prescribing (0.64) and the lowest were for antibiotic prescribing (0.42) and injec-tion use (0.38) (Table 4). The Spearman's correlation coefficient for the number of medicines prescribed as generics versus cost was 0.22 (p < 0.01), indicating that there is a weak positive relationship between these two variables. The Spearman's correlation coefficient for the number of medicines prescribed from the NEML and cost was 0.19 (p < 0.01), indicating that there is also a very weak positive relationship between these two variables also. The average cost of medicines per prescription is Le. 29,376.30 ($ 6.78) [22] (Table 5). At the time of the study, a person would have to work for about 43 days (based on the minimum wage of the lowest paid government worker) or about 5 days (based on the average negotiated minimum wage) [23] to be able to pay for the medicines prescribed.

**Table 2:** Demographic data of outpatients seen at the selected hospitals

	Number	Percentage
**Age**		
6 – 20 years	133	22.1
21 – 40 years	245	40.8
41 – 60 years	121	20.2
61 – 80 years	56	9.3
Above 80 years	7	1.2
Adult*	38	6.4
**Sex**		
Male	282	47.0
Female	318	53.0

Adult *- on few prescriptions, the exact ages of patients were not recorded, but simply written as adult

**Table 3:** WHO prescribing indicators at the four hospitals individually and overall

	Hospital A	Hospital B	Hospital C	Hospital D	Overall results
Number of medicines prescribed per encounter	Mean 3.56 (SD 1.35)	Mean3.3 (SD 1.10)	Mean 6.66 (SD 3.18)	Mean 3.93 (SB 1.60)	Mean 4.37 (SD 2.39)
Percentage of drugs prescribed by generic name	50.8	75.3	60.4	42.3	57.3
Percentage of encounters when an antibiotic was prescribed	59.3	73.3	92.7	60	71.5
Percentage of encounters when an injection was prescribed	18.7	18.7	58.7	8	26
Percentage of medicines prescribed from the NEML	52.8	84.1	67.8	50.2	63.9

**Table 4:** Index of Rational Drug Prescribing (IRDP)

Hospital	Index of poly-pharmacy	Generic prescribing index	Index of rational antibiotic prescribing	Index of rational prescribing of injectable medicines	Essential medicines Prescribing index	Index of Rational Drug Prescribing (IRDP)
A	0.75	0.51	0.51	0.53	0.53	2.83
B	0.87	0.75	0.33	0.53	0.84	3.32
C	0.32	0.60	0.32	0.17	0.68	2.09
D	0.60	0.43	0.50	1	0.50	3.03
**Overall**	0.64	0.57	0.42	0.38	0.64	2.65

* IRDP – Index of Rational Drug Prescribing for each hospital (maximum value =5)

**Table 5:** Cost and affordability of a prescription

Hospital	CH(CI)	LGH	MSGH	RGH	Overall results
Average cost of a prescription in Leones (and US dollars)	36, 067 ($ 8.32)	10, 670 ($ 2.46)	39, 310 ($9.07	30, 630 ($ 7.07)	29,376 ($ 6.78)

## Discussion

The average of 4 medicines (range 3-7) per prescription (Table 2) suggests polypharmacy occurs in some instances. Although not synonymous, polypharmacy and inappropriate prescribing are strongly associated [24] and can result in drug interactions, adverse drug reactions, non-adherence to therapy, medication errors and increased treatment costs [11]. This mean of 4 medicines may reflect a slight increase from 2.7 over the past fifteen years in the number of medicines prescribed per patient by doctors in Western Sierra Leone, although the populations may not be directly comparable [19]. It is similar to values obtained from the West African sub-region where the average number of medicines per prescription was 4 in Liberia [25], 3.5 and 3.7 in two studies in Ghana [26, 27] and 3.55 and 3.9 in Nigeria [28, 29]. The use of unnecessarily expensive drugs when cheaper effective alternatives are available is one of the characteristics of irrational prescribing [6]. Thus, generic prescribing is being promoted as a key component of rational medicines use. The overall average of 57% may indicate some improvement in generic prescribing from 45% fifteen years ago [20] and does not differ greatly from generic prescribing trends in Warri, Southern Nigeria (54%) [29], and the Ghana Police Hospital(62.6%) [27]. Globally, generic prescribing continues to be a challenge, evident from the low generic prescribing rates in the Manipal Teaching Hospital, Nepal (13%) [30], Sao Paolo Brazil (30.6%) [31], in Lim-popo and Western Cape provinces, South Africa (45.2%) [32] Montserrado county, Liberia (38%) [25] and Umtata, South Africa, where only 10.4% of prescriptions for antimicrobials were written using generic names [33].

Antibiotics are one of medicine's greatest achievements [34] resulting in a dramatic fall in morbidity and mortality associated with bacterial infections over the last fifty years [4]. Their indiscriminate use however results in antibiotic resistance and serious consequences. Appropriate use of an-timicrobials is defined as their cost-effective use, which maximizes clinical therapeutic effect while minimizing drug-related toxicity and the development of antimicrobial resistance [35]. With antibiotic prescribing at outpatient departments of government hospitals in Freetown being 76% (Table 2), there is a possibility of an increased risk of resistance. For comparative purposes, 83% of all prescriptions in a study in neighbouring Liberia involved an antibiotic [25], but in a Nigeria, antibiotic prescribing occurred in 28% [36] 49.2% [28] and 56% of encounters [29]. In a na-tional survey of the pharmaceutical sector of Ghana, the result for this indicator was recorded as 43% [26]. In southern Ethiopia and Khartoum, Sudan, an antibiotic was prescribed during 58.1% [37] and 65% [38] of patient encounters respectively. In the Eastern province of Saudi Arabia, encounters with an antibiotic prescribed were 32.2% [26] and in Western China, the percentage of prescriptions containing antibiotics were 48.4% [16]. Injections are the most common health care procedure worldwide and in developing and transitional countries alone, some sixteen thousand million injections of medicines are administered annually [38], up to 70% of which are however believed to be unsafe [14]. Although overall, 26% of pre-scriptions included a medicine by injection (Table 2), and prescribing by injection had the lowest overall index, injection prescribing was lower than recommended (<10%) at one hospital. In Mont-serrado county, Liberia and at a teaching hospital in southern Ethiopia, 26.2% [25] and 38.1% [37] of patients received medicines by injection respectively. Lower values were recorded in Lagos, Nigeria, where mean prescribing of injectable medicines was 5.8% [30] and in public hospitals in South Africa, 8.3% [34]. Reasons for unnecessarily prescribing injections include trying to satisfy patient demands and some prescribers' perception that injections provide faster relief from symptoms and greater clinical effectiveness [39].

Essential medicines lists promote the use of a minimum number of selected medicines with the in-tention of ensuring their more rational use at the least possible cost to the health system and consumers [15]. Over 60% of the medicines prescribed at the hospitals mentioned were from the NEML (Table 2), an improvement from the 25% recorded fifteen years ago [19]. The NEML was not available at the outpatient departments of any of the hospitals which may account for the suboptimal percentage of medicines prescribed from it. Doctors will not consciously prescribe from a list with which they are not familiar. In Nepal, 32.8% [30] and at clinics in Western China 67.7% of medicines [16] were prescribed from their National Essential Drug Lists. At the Ghana Police Hospital, 53.6% of drugs prescribed were on the hospital's EML [27]. Developing a National Essential Medicines List (NEML) is crucial in the promotion of rational prescribing, but its value is contingent upon ensuring that it is accepted and used by prescribers [40]. The overall IRDP of 2.65 is considerably lower than the ideal of 5, which indicates that prescribing at outpatient departments of government hospitals in Freetown Sierra Leone could be improved fur-ther. The IRDP of these government hospitals in Freetown was lower than that obtained from some other studies. From a study of rational prescribing in Tanzania, the overall IRDP was 3.8 and that of facilities in urban areas like the ones in this study was 3.42 [41]. In Western China, the IRDP was 3.32 ranging from 3.12 to 3.98 across 10 selected provinces [16]. At 10 health care centres in the Eastern province of Saudi Arabia, the IRDPs ranged from 3.77 to 5 [42].

Medical insurance schemes in Africa may not cover prescription medicines on an outpatient basis [3] and thus the cost of a prescription is one barrier to accessing optimum healthcare. At one hospital in this study which had the highest average cost per prescription, there was a high rate of generic prescribing, which should result in a comparatively low cost for each item, but this was offset by the large amount of individual medicine items (an average of 6.7) per prescription. At two of the hospitals, the number of medicines prescribed per patient was much less, but due to less generic prescribing, the average cost of prescriptions was also high. At one hospital, there were fewer medicine items in each prescription and a high rate of generic prescribing, which might ex-plain the much lower cost of a prescription. This indicates that although excessive prescribing by brand names and minimal prescribing from the EML tends to increase the cost of a prescription, the number of medicines prescribed may be the stronger determinant of the total cost to the patient. Al-lied to this, over half of the population of Sierra Leone (53.37%) live on less than 1.25 US dollars per day [8]. In Nepal, the average cost per prescription was Nepalese Rupees 285.99 (US $ 3.73) [32], about half the average cost of a prescription at government hospitals in Freetown. In India, the cost of treatment for community acquired pneumonia required one to three days work by a daily wage earner, depending on the brand of medicine prescribed [43]. Around 72% of the total health expenditure in Sierra Leone is from private funds, 57.3% of which are out-of-pocket. Thus, out-of-pocket payments are the primary means of paying for healthcare for most Sierra Leoneans, especially those visiting government hospitals [8]. Inequity may arise if access to care is strongly influenced by affordability [20]. Therefore, necessary medicines may not be purchased, or may be bought in insufficient quantities [3]. Although the minimum wage of a government worker has been recently increased [44], affordability may still remain a problem.

This study provides baseline information on prescribing patterns at government hospitals in Free-town and further attempts to highlight the extent of affordability of medicines by patients resulting from doctors' prescribing. This is necessary for building any interventional framework and for future audit purposes. However, the factors influencing the prescribing practices of these doctors, such as level of training and experience were not elucidated. In addition, the appropriateness of antibiotic prescribing was not elevated as the indications for antimicrobial use was not captured in this study. Also, a full pharmaco-economic assessment of cost of care was not conducted, as other direct medical and non-medical and indirect costs would need to be calculated. Prescribing at private hospitals was not evaluated. Further studies addressing the above limitations of this study issues would be valuable.

## Conclusion

We conclude that prescribing by doctors at government hospitals in Freetown, especially in regard to antibiotic and injection prescribing, can be rationalised to be more cost-effective whilst maintaining clinical effectiveness. It is also evident that for many Sierra Leoneans, prescriptions for medicines are very expensive, so it is important to ensure that rational prescribing is optimised in order to obtain maximum value for money.

### What is known about this topic

- Rational medicines use remains a challenge worldwide and involves maximizing clinical and cost-effectiveness. Assessing adherence to quality prescribing standards is integral to ensuring rational medicines use;
- The only published study on prescribing patterns in adults in Sierra Leone was by D. Lisk and L. Palmer in 1997;
- In low- and middle-income countries, many patients cannot afford their prescriptions, in part because out-of-pocket payments are the main source of health care financing.

### What this study adds

- This study provides baseline data on prescribing patterns at four government hospitals in Sierra Leone 15 years after the study by Lisk and Palmer. We also assessed the extent of rational prescribing using the Index of Rational Drug Prescribing (IRDP);
- We have also evaluated the affordability of such prescriptions, providing an assessment of how prescribing affects patients;
- The study shows that improvements in rational prescribing are still possible and would contribute to the affordability of medicines for individual patients.

## Competing interests

The authors declare no competing interests.
